# Supplementary figures and images for: Placenta Transcriptome Profiling in Intrauterine Growth Restriction (IUGR)
Source: Int J Mol Sci. 2019 Mar 26;20(6):1510. doi: 10.3390/ijms20061510 (PMC6471577; doi:10.3390/ijms20061510)

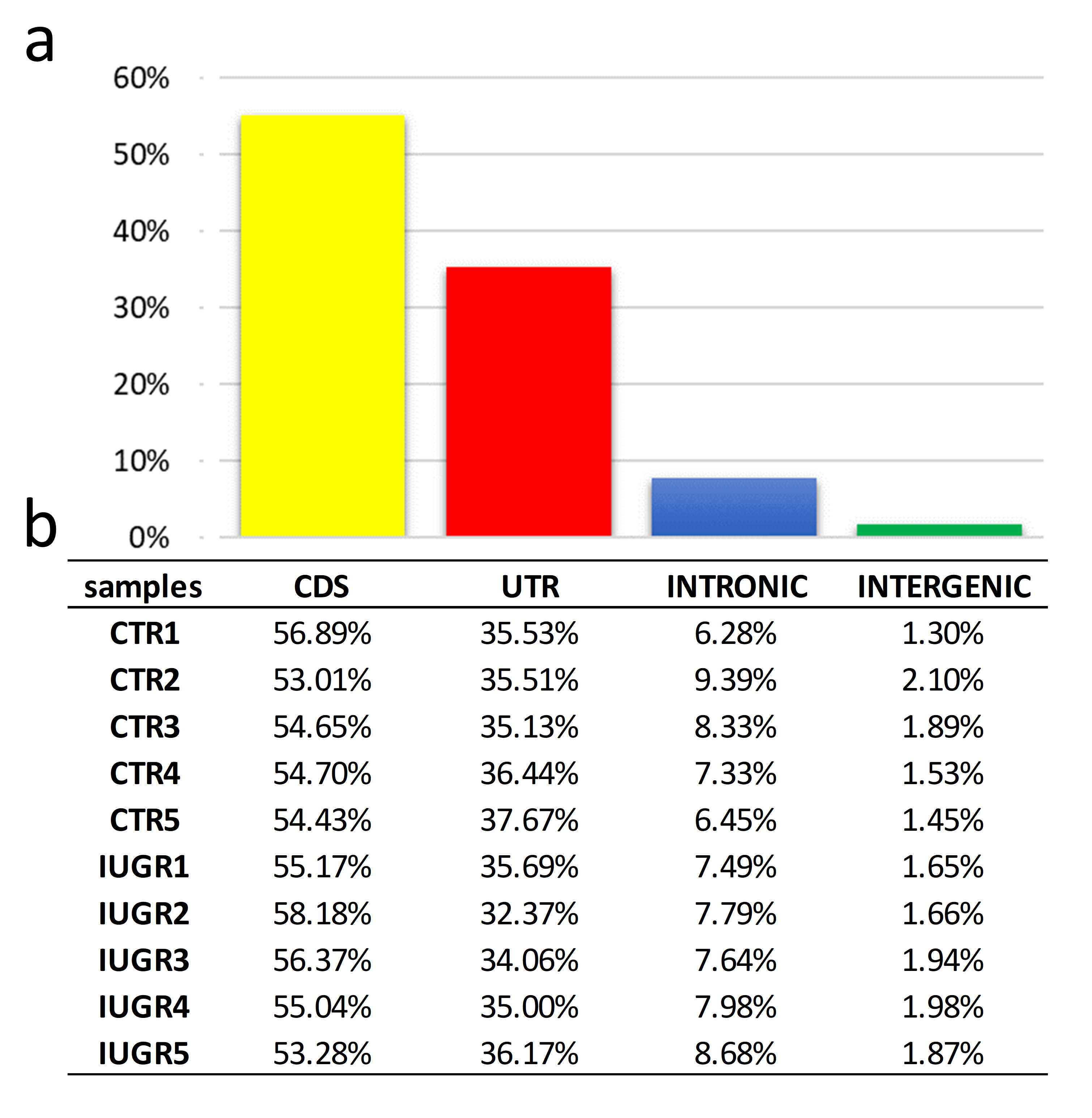

Supplement: Supplementary file 1 [file ijms-20-01510-s001.zip › Fig.1S Majewska et al..tif]
